# The management dilemma of a large left ventricular fibroma

**DOI:** 10.1093/ehjcr/ytaf644

**Published:** 2025-12-11

**Authors:** Michael Gomes, Denae Moore, Wasing Taggu

**Affiliations:** Joondalup Health Campus, Shenton Avenue, Joondalup, WA 6027, Australia; Royal Darwin Hospital, 105 Rocklands Drive, Tiwi, NT 0810, Australia; Joondalup Health Campus, Shenton Avenue, Joondalup, WA 6027, Australia

**Keywords:** Case report, Fibroma, Echocardiography, Multimodality imaging, Tumour, CT, MRI

## Abstract

**Background:**

Cardiac fibromas are rare, benign primary cardiac tumours predominantly observed in paediatric populations, with their occurrence in adults being exceptionally uncommon.

**Case summary:**

We present the case of a 40-year-old physically active male with an incidentally discovered, large left ventricular fibroma during investigation for presumed ischaemic heart disease. Multimodal imaging, including computed tomography (CT) coronary angiography, transthoracic echocardiography, cardiac magnetic resonance imaging, and positron emission tomography-CT, revealed a 42 × 30 × 58 mm myocardial mass with significant calcification and myocardial invasion, ultimately diagnosed as a fibroma via biopsy. Given the patient’s asymptomatic status, frequent ventricular ectopics, and high surgical risk associated with resection, a conservative management strategy was adopted. This included regular Holter monitoring and echocardiography to assess for arrhythmias, tumour progression, or functional compromise. The case underscores the limited evidence available for managing cardiac fibromas in adults, necessitating extrapolation from paediatric data and an individualized, patient-centred approach.

**Discussion:**

This report highlights the challenges of decision-making in adult cardiac fibromas, particularly regarding arrhythmogenic potential and surgical considerations, and emphasizes the need for further studies to establish evidence-based guidelines for this rare condition.

Learning points Primary

- Cardiac fibromas are rare benign tumours but can be arrhythmogenic. This must be considered when choosing a management strategy.
- Serial monitoring for arrhythmias and tumour growth is important to prevent complications and aid in decision-making.

Secondary

- Multimodality imaging is a useful tool in evaluating the aetiology of cardiac masses.
- The management must involve shared decision-making and a patient-centred approach.

## Introduction

Cardiac fibromas, although rare,^1^ represent a significant clinical entity in both paediatric and adult populations. These primary cardiac tumours, predominantly composed of fibrous tissue, are more commonly diagnosed in children, where they are the second most frequent type of cardiac tumour.^2^ In adults, their occurrence is notably rarer, rendering each case a unique clinical challenge. Cardiac fibromas are typically benign, but can have serious implications such as mechanical complications and life-threatening arrhythmias.^2,3^ They often present asymptomatically and are incidentally found, especially in adults, as presented in this case.

The management of cardiac fibromas remains a complex decision-making process, primarily due to the risk of life-threatening arrhythmias such as ventricular tachycardia (VT), which has been found in up to 31% of patients in small adult case series.^1^ This arrhythmogenic potential necessitates careful consideration in both surveillance and intervention strategies. The risk of VT has been much better defined in paediatric populations with up to 64% experiencing VT.^4^ The current evidence for management is largely derived from case reports, with surgical resection the preferred approach in symptomatic individuals or those at risk of complications. However, the management strategy in asymptomatic adults or those with high surgical risks—due to location and size of the tumour—is less clear-cut and often pursue surveillance management. This highlights the need for individualized patient-centred care.

We present a case of an incidentally found large left ventricular fibroma abutting the myocardium and causing frequent ventricular ectopics. At presentation, the patient likely had no related symptoms, leading to a dilemma in pursuing either a conservative or surgical approach.

## Summary figure

**Table:** 

Day 1	Epigastric pain
	Presented to nursing post
	Examination, electrocardiogram (ECG), and bloodwork
Day 7	Computed tomography coronary angiogram (CTCA)
Day 9	Transthoracic echocardiogram (TTE)
Day 12	Cardiac magnetic resonance imaging (MRI)
Day 13	PET/CT (F-18 FDG)
Day 14	Results explained to patient and referred to cardiac centre
Day 45	Biopsy via left anterior mini-thoracotomy, suggesting fibroma
Day 120	Increasing burden of ventricular ectopy detected by Holter monitor (2050/day) and one episode of non-sustained VT. Bisoprolol commenced. Stable size on echocardiogram
Day 210	Repeat Holter demonstrated ongoing ventricular ectopy (977/day)
Day 245	Loop recorder inserted

## Case presentation

**Figure 1:**
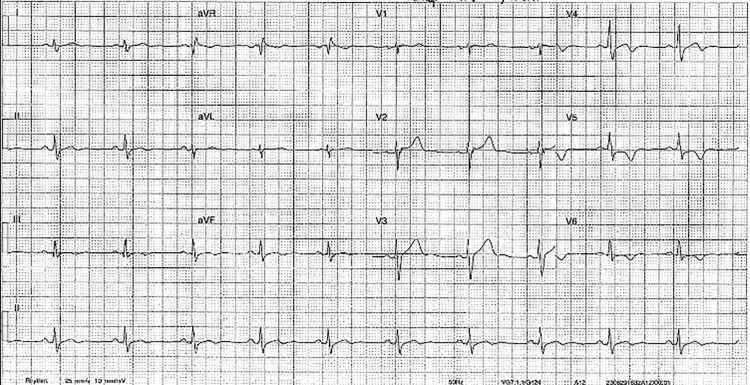
Twelve-lead electrocardiograph on initial presentation, showing sinus rhythm with T wave inversion in inferior and lateral leads.

A 40-year-old physically fit and active man, during a camping trip with his family, developed a persistent, aching epigastric pain, with no exertional component. He had a past medical history of gastro-oesophageal reflux disease (GORD) and anxiety, taking 20 mg of escitalopram daily. He presented to a remote nursing post, where his initial evaluation included an unremarkable physical examination, blood tests including negative high-sensitivity cardiac troponins, and serial ECGs. The ECGs demonstrated sinus rhythm but with notable dynamic deep T wave inversion in the inferior and lateral leads (*Figure 1*). Additionally, regular ventricular ectopic beats were noted on subsequent ECGs. With observation time and resolution with antacids, the impression of the pain was that of GORD. However, the unusual ECG findings prompted referral for further investigation.

**Figure 2:**
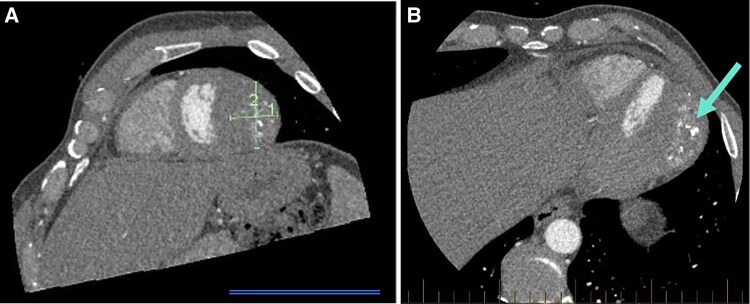
Computed tomography coronary angiogram, axial images displaying a 42 × 30 × 58 mm cardiac mass (*A*). (*B*) The complexity of the lesion with areas of calcification (blue arrow) and demonstrating the difficulty delineating whether arising from pericardium or myocardium.

**Figure 3:**
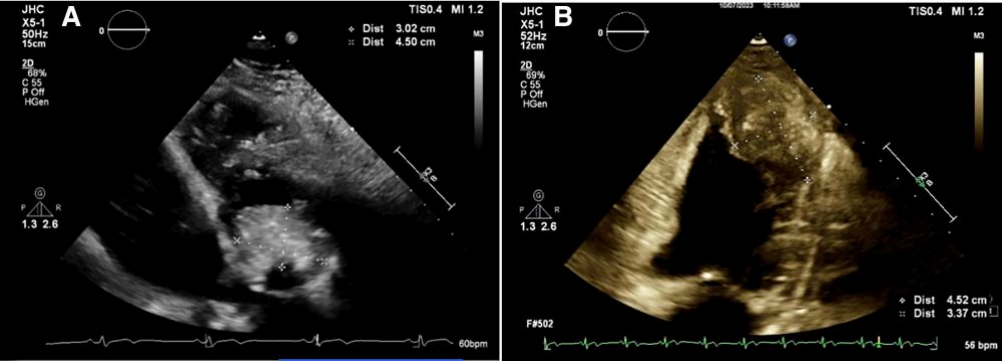
Transthoracic echocardiogram images. A flipped apical four-chamber view (*A*) demonstrating a 3.02 cm by 4.5 cm well-circumscribed mass within the apical pericardium. (*B*) An apical two-chamber view with a mass of 4.52 cm × 3.37 cm.

Following review with a cardiologist, a CTCA and TTE were ordered first to investigate for coronary artery disease. While the coronary arteries were unremarkable, the CTCA revealed a significant extracoronary finding of a localized soft tissue mass, measuring 42 × 30 × 58 mm. It was observed lateral to the mid to distal third of the left ventricular lateral wall, demonstrating dystrophic calcification and having a mass effect with indentation of the wall (*Figure 2*). The TTE identified the large, well-circumscribed mass thought to be within the apical pericardium, potentially indicative of a tumour or thrombus (*Figure 3*). The left ventricle (LV) was of normal size with no regional wall motion abnormalities and preserved systolic function (ejection fraction 57%). A severely dilated left atrium was noted but otherwise, normal diastolic function, and no significant valvular dysfunction.

**Figure 4:**
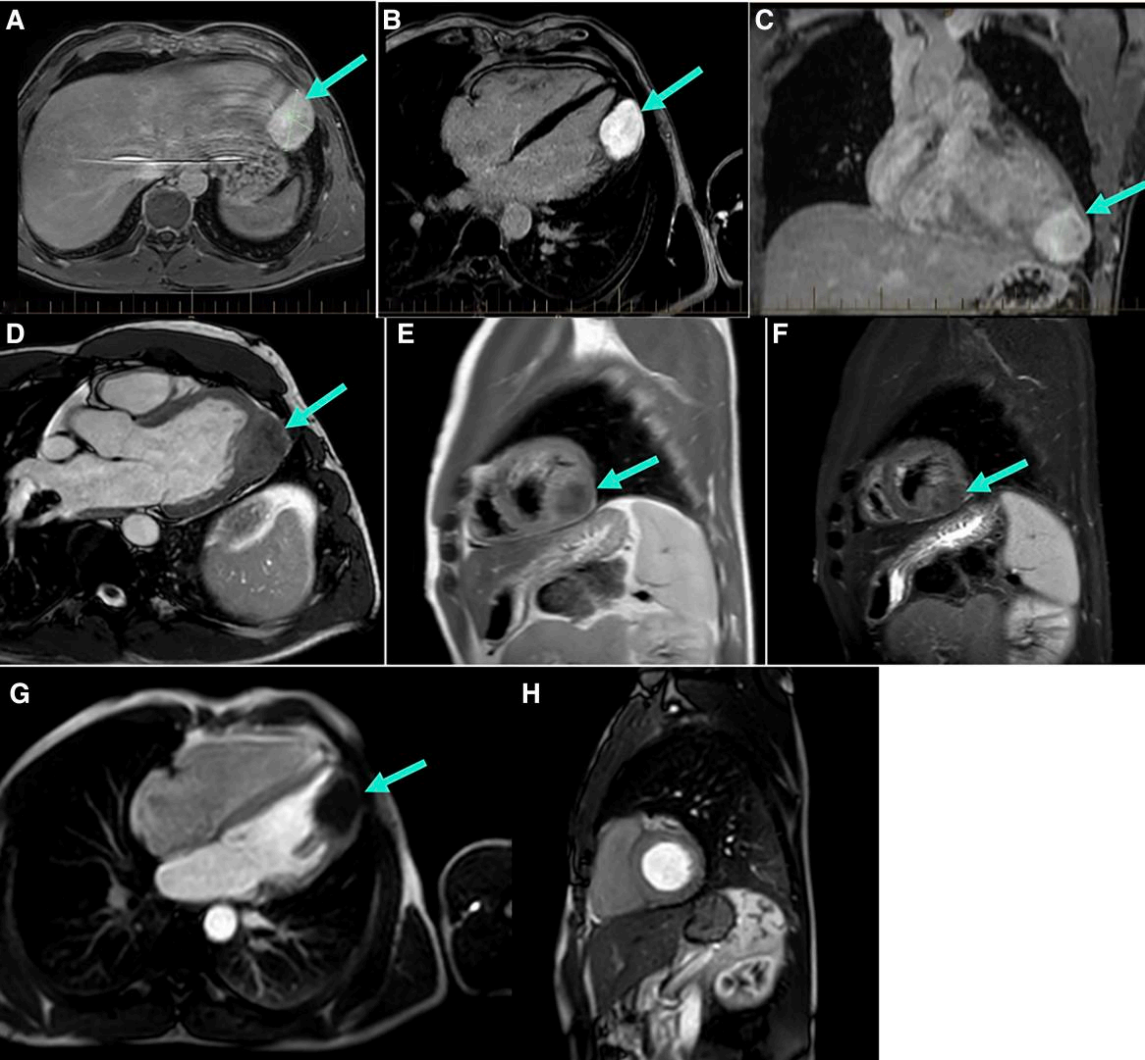
Cardiac magnetic resonance imaging demonstrating the morphological and tissue characteristics of the left ventricular mass. Axial (*A* and *B*) and coronal (*C*) late gadolinium enhancement images show a well-circumscribed intramyocardial mass with intense, homogeneous delayed enhancement (blue arrows). T1-weighted (*D* and *E*) images show the lesion as isointense to mildly hypointense relative to normal myocardium, while T2-weighted short tau inversion recovery imaging (*F*) demonstrates low T2 signal intensity, consistent with dense fibrous tissue. First-pass perfusion sequences (G: axial; H: sagittal) show absent enhancement within the mass, confirming its avascular nature. The overall pattern of low T2 signal, homogeneous intense late gadolinium enhancement, and absent first-pass perfusion is characteristic of a cardiac fibroma, distinguishing it from other cardiac tumours such as myxoma, rhabdomyoma, or sarcoma.

**Figure 5:**
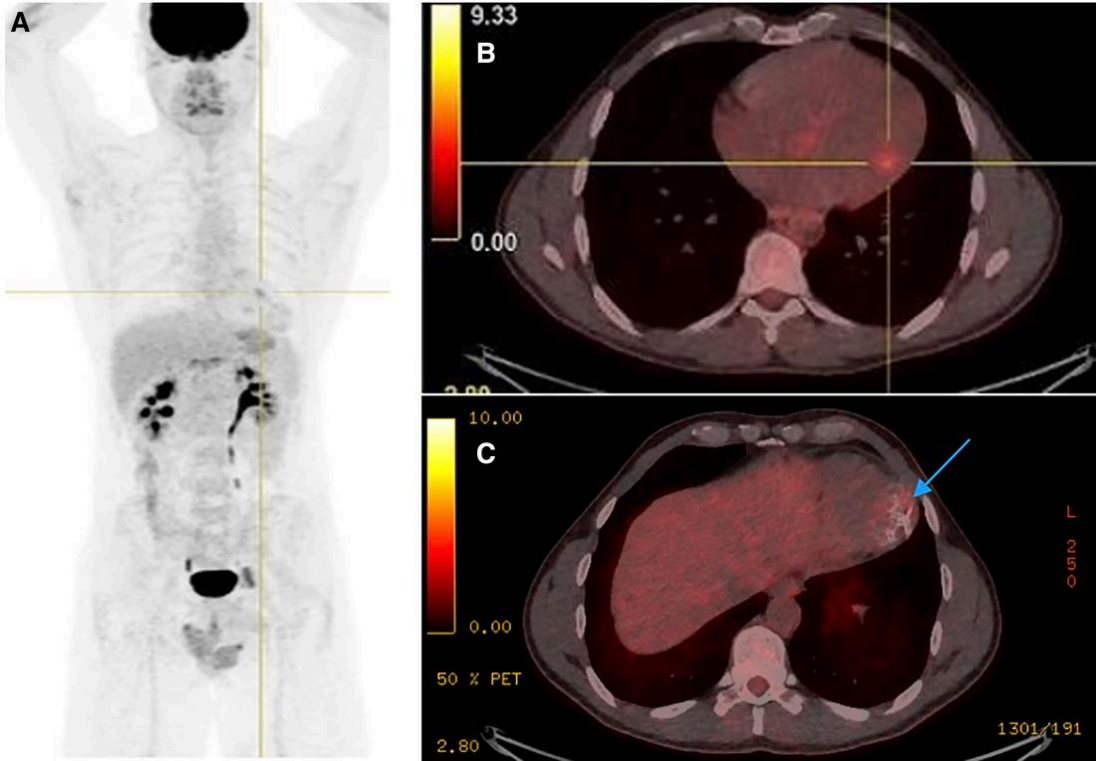
(*A*) Fluorodeoxyglucose F-18 positron emission tomography (PET), coronal view showing physiological distribution of fluorodeoxyglucose F-18. (*B*) Positron emission tomography-computed tomography axial view demonstrating moderate uptake localizing to a normal papillary muscle (presumed physiological). (*C*) No evidence of pathological uptake throughout the myocardial mass (blue arrow).

To further characterize the mass and delineate the aetiology, cardiac MRI was requested. It found that the mass was centred in the myocardium rather than the pericardium or pleura, extending from the apical to mid portion of the lateral to inferolateral LV wall. On T1-weighted MRI, it was isointense to mildly hypointense, and on T2-weighted MRI, it was markedly hypointense, and it showed avid homogeneous delayed enhancement with gadolinium (*Figure 4*). Furthermore, on first-pass perfusion sequences, there was no enhancement supporting its avascular nature. All these features are indicative of a fibrous tumour, and at this stage, other differentials including thrombus, lipoma, rhabdomyoma, and sarcoma were excluded. To support the diagnosis and to ensure it was not malignant, positron emitting topography (F-18 FDG PET-CT) was performed (*Figure 5*). This showed physiological uptake throughout the body, presumed physiologic uptake of papillary muscle in the heart, and no abnormal uptake in the cardiac mass consistent with a fibroma.

After all these initial images were performed, the patient was referred to a quaternary cardiac centre, where the patient underwent a left anterior mini-thoracotomy for biopsy. This suggested a fibroma, given the appearance of spindle fibroblasts in a dense collagenous stroma. A multidisciplinary team (MDT) meeting was held, and given the mass’s significant size and location, surgical resection was deemed high risk due to the potential of leaving insufficient myocardium for functional LV integrity.

A conservative approach was chosen due to the incidental discovery and absence of overt symptoms. This included regular interval Holter monitoring to monitor arrhythmias and transthoracic echocardiograms to monitor any growth and assess its impact on LV function. Over the following 6 months, the Holter monitors demonstrated an increasing burden of uniform ventricular ectopy (up to 2050/day) and one episode of non-sustained VT lasting 14 beats at a rate of 134 b.p.m. Thus, bisoprolol was commenced, aiming to suppress the ectopy and reduce the chance of sustained ventricular arrhythmias. A loop recorder was inserted to provide further monitoring.

At another MDT clinicians discussed that should interval change in the mass occur, significant symptoms or significant arrhythmias, like recurrent non-sustained VT, then further intervention will need to be considered. For example, implantable cardioverter-defibrillator (ICD) implantation is considered in our centre if there is haemodynamic instability with recurrent non-sustained VT or certainly sustained VT as per the ESC guidelines.^5^ The loop recorder may provide sufficient evidence for this to be considered in this patient. Anti-arrhythmic medication to supress simple ectopics would be used first, with ICD being a last line. Cryoablation could be considered for VT if monomorphic and deemed possible by electrophysiologists. Finally, if the mass is demonstrated to be increasing in size in interval imaging or causing mechanical issues, resection, or even transplant will be considered by the multidisciplinary team.

## Discussion

The aforementioned case deals with complex decision-making, with minimal evidence and guidelines to inform clinicians. Cardiac tumours are rare, and adult fibromas are rarer still, with most of the evidence presented in the paediatric population and extrapolated to adults. Firstly, surgical resection is deemed the recommended approach due to the arrhythmogenic potential of cardiac fibromas in this population, including ventricular tachyarrhythmias.^2^ One multicentre study with 29 paediatric patients identified 62% having sustained VT and 23% suffering from cardiac arrest while awaiting surgery.^4^ In comparison, a recent case series involves 26 patients, 14 of which were adults with cardiac fibromas, 23% had non-sustained VT, and 12% had sustained VT.^1^ This highlights some potentially lower rates of VT in adults and necessitates more research into adult cardiac fibroma complications.

Regarding management choice, particularly in asymptomatic patients, it is important to evaluate the surgical risk of resection. As in some cases, like the one presented, there may be proximity to the myocardium and resection would not leave sufficient, viable myocardium to form a functional LV. A temporizing measure describes simple anti-arrhythmic to decrease burden of ectopy if suitable. Another option less invasive than surgery for unresectable tumours is cryoablation. One electrophysiological study looked at cryoablation around the edges of cardiac tumours to supress tumour related VT and summarized it as a promising therapeutic option.^6^ A further management option to reduce the risk of death from malignant arrhythmias is the use of an ICD. There are very few case reports on the use of cardiac defibrillators for unresectable fibromas; however, it is used in a few case reports.^1,7,8^ A recent case study described a 55-year-old man be managed with an ICD for frequent ectopics, although he also had a family history of sudden cardiac death.^7^ There is currently very minimal evidence to support timing and choice of intervention for these patients with most evidence in the form of case studies.

If symptoms are concerning, interval growth or malignant arrhythmias are apparent; then surgical options would be reconsidered in the presented patient. One option that could be considered is a partial resection, which is documented as an approach undertaken in a few case series with large fibromas.^1,9^ However, if following this arrhythmogenic substrate remains, it leaves orthotropic heart transplant as the remaining therapeutic option. This has only been performed for this indication in paediatric populations, with an age range spanning 1 month old up to 17 years old, and this case series found reduced long-term survival when comparing heart transplant to surgical resection, even in large masses.^9^

Evidently, a consensus on management in adults is lacking in current research. A recent case series from the Mayo Clinic looked retrospectively at cardiac fibromas diagnosed between 1964 and 2020 and found 26 including 14 adults and 12 paediatrics.^1^ While 100% of the paediatrics underwent surgical resection, only 50% of the adults did.^1^ The consensus of paediatric patients undergoing resection is likely due to the higher risk of mortality in this age group.^1^ In the adults who were managed with surveillance, at follow-up (ranging from 10 months to 12 years), four adults were still asymptomatic, two had palpitations/non-sustained VT, and one was lost to follow-up. This is supported by another recent case series of three patients, each with surveillance management pursued, including one having arrhythmias and getting an ICD.^7^ More research is needed to justify, firstly which patients are suitable for surveillance management, what follow-up is needed for these patients, and detailed criteria for when intervention is justifiable.

In conclusion, there are very few large studies to guide clinical decision-making for large adult cardiac fibromas. There is a consensus regarding preference for surgical resection in current literature; however for high risk, large fibromas, there are more conservative surveillance strategies being implemented based on recent case reviews. Evidently, a multidisciplinary team discussion is needed for each individualized case to determine the best management option. Surveillance with echocardiogram and loop monitoring is the current management strategy for the presented patient; however with further research, it will make decisions on management in patients like this more evidence based with hopefully consensus guidelines available in the future.

## Patient’s perspective

The patient is a very fit and active man, previously running 10 km and swimming 1 km, while almost completely asymptomatic with a few minor GORD-related symptoms. Despite permission to exercise, the awareness of his condition brought about some uncertainty and consequential psychosomatic barriers. He is understandably anxious for the future, given the nature of the large fibroma and the potential risk of arrhythmias. The effects also extend to family members and their anxieties surrounding their loved one and the unknown. However, he is determined to not let it impact his life significantly and has resumed work, albeit in a different field, and is pursuing exercise.

## Lead author biography


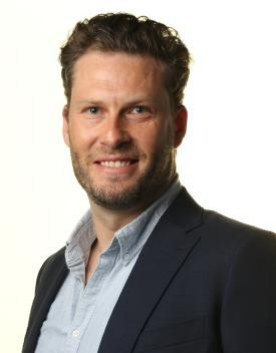


Michael Gomes, MBBS, is a medical registrar and chief registrar at Royal Darwin Hospital, Northern Territory, Australia. He completed his medical degree at the University of East Anglia, UK, and has worked across tertiary and regional centres in both the UK and Australia. Michael has a strong academic interest in cardiology, with experience in complex cardiac imaging and structural heart disease. He is also completing a Master of Medicine (Internal Medicine) through the University of Sydney, with a focus on clinical research and is pursuing advanced training in cardiology. He is particularly interested in imaging and in future wishes to pursue a career in sports cardiology.

## Data Availability

The data underlying this article are available in the article and in its online supplementary material and were provided to the corresponding author with permission of Joondalup Health Campus.
